# Robotic stereotactic radiosurgery for intracranial meningiomas in elderly patients: assessment of treatment efficacy and safety

**DOI:** 10.3389/fonc.2024.1329696

**Published:** 2024-01-29

**Authors:** Anton Früh, Bohdan Bodnar, Marcel Nachbar, Julia Gradhand, Goda Kalinauskaite, Kerstin Rubarth, Peter Truckenmueller, David Kaul, Daniel Zips, Peter Vajkoczy, Carolin Senger, Güliz Acker

**Affiliations:** ^1^ Department of Neurosurgery, Charité - Universitätsmedizin Berlin, Corporate Member of Freie Universität Berlin, and Humboldt-Universität zu Berlin, and Berlin Institute of Health, Berlin, Germany; ^2^ Berlin Institute of Health Charité Junior Digital Clinician Scientist Program, Berlin Institute of Health Biomedical Innovation Academy, Berlin, Germany; ^3^ Department of Radiation Oncology, Charité - Universitätsmedizin Berlin, Corporate Member of Freie Universität Berlin, and Humboldt-Universität zu Berlin, and Berlin Institute of Health, Berlin, Germany; ^4^ Insitute of Biometry and Clinical Epidemiology, Charité - Universitätsmedizin Berlin, Corporate Member of Freie Universität Berlin, and Humboldt-Universität zu Berlin, and Berlin Institute of Health, Berlin, Germany; ^5^ Insitute of Medical Informatics, Charité - Universitätsmedizin Berlin, Corporate Member of Freie Universität Berlin, and Humboldt-Universität zu Berlin, and Berlin Institute of Health, Berlin, Germany; ^6^ Berlin Institute of Health at Charité – Universitätsmedizin Berlin, Berlin, Germany

**Keywords:** meningioma, elderly patients, CyberKnife, stereotactic radiosurgery, brain tumor

## Abstract

**Purpose:**

Stereotactic radiosurgery (SRS) has been increasingly used to treat intracranial pathologies in elderly patients. The treatment efficiency of SRS has been demonstrated in meningiomas, with excellent local control. We aimed to analyze the safety of robotic SRS in elderly patients with meningiomas.

**Methods:**

We searched for patients with suspected WHO °I meningioma ≥ 60 years old, who underwent CyberKnife (CK) SRS from January 2011 to December 2021. Tumor localization was categorized using the “CLASS” algorithmic scale. Tumor response was evaluated using the Response Assessment in Neuro-Oncology (RANO) criteria for meningiomas. Adverse effects were graded using the Common Terminology Criteria for Adverse Events (CTCAE) version 5.0 and a cox regression was performed to investigate possible predictors.

**Results:**

We identified 82 patients with 102 CK-treated lesions that matched the criteria for the first SRS. The median age was 70 [IQR 64-75] years, and 24.3% of the patients were aged > 75 years. Multiple lesions (up to six) were treated in 14.1% of the SRS-sessions. A previous surgery was performed in 57.3% of lesions, with a median time interval of 41 [IQR 10 – 58] months between the initial surgical procedure and the SRS treatment. In 47.9% of cases, CLASS 3 meningiomas at high-risk locations were irradiated. Single fraction radiosurgery was applied to 62.5% of the lesions, while in the remaining cases multi-session SRS with three to five fractions was used. During the median follow-up period of 15.9 months, lesion size progression was observed in 3 cases. Karnofsky Performance Status (KPS) declined by ≥ 20 points in four patients. Adverse effects occurred in 13 patients, while only four patients had CTCAE ≥2 toxicities. Hereby only one of these toxicities was persistent. The occurrence of complications was independent of age, planned target volume (PTV), high-risk localization, and surgery before SRS.

**Conclusion:**

The data indicates that SRS is a safe, efficient, and convenient treatment modality for elderly patients with meningioma, even at high-risk locations

## 1 Introduction

Meningiomas arise from the cap cells of the arachnoid membrane (1, 2) and are the most common primary tumors of the brain (3–6). From a rate of 0.1 in children to 27.8 in the 75-to-84-year age group, the incidence of meningiomas per 100,000 people rises with age, with women being more affected than men (6, 7). The clinical presentation is location dependent. Most cases are diagnosed as asymptomatic, small, slow-growing tumors without brain edema (2, 8, 9). If meningiomas become clinically symptomatic, increase in size or are located at a site that may lead to neurological symptoms, treatment is required (2). Currently, no effective drug therapies are available (3). Therefore, surgery remains the gold standard for these lesions. In addition to removing the growing mass, operative therapy allows for histopathological diagnosis, reduces neurological symptoms, and is associated with good long-term tumor control (2, 10). For smaller lesions radiosurgery and observation are therapy options. While no high-level evidence exists, local control rates after treatment with SRS are very high at 86-100 % (11). Furthermore, a recent trial examining vestibular schwannomas found an advantage concerning local tumor growth (12).

Owing to an aging society, improved life expectancy and advanced diagnostic tools, incidence in elderly patients will increase with time, and clinicians will encounter a larger population of older patients (13–15). Due to the presence of diverse comorbidities and limitations of physiological capacities, the management of these patients remains challenging (2, 14). Therefore, surgical therapy in elderly patients with meningiomas is more beneficial than wait-and-see strategies but it results in higher morbidity and mortality rates compared to younger patients (15, 16). Thus, balancing the potential risks of surgery with alternative non-invasive procedures, such as stereotactic radiosurgery (SRS), is important, especially in patients with small intracranial lesions in complex locations. SRS is an advanced technique that enables highly precise radiation with high single doses for treating brain, spine, and other tumors in the body (17, 18). This treatment is often performed in a single session. However, hypofractionated (multi-session) treatments are possible for larger lesions or to protect critical structures (19). With regard to intracranial applications, studies have shown that SRS has comparable efficacy to conventional radiation therapies and is associated with fewer side effects (20, 21). The efficacy of SRS treatment has also been demonstrated for benign brain tumors, such as meningiomas, with adequate local control and a satisfactory incidence of side effects (2, 14). Adequate efficacy with high safety, shortened treatment duration, and the resulting decrease in patient burden, especially due to outpatient therapy in the clinic, makes SRS the treatment of choice when technically feasible. Therefore SRS represents a convenient treatment option for elderly patients. Consequently, SRS has been increasingly used to treat intracranial pathologies, such as metastases, also in elderly patients (14, 22–25). In this single-center study, we critically investigated the feasibility and safety of radiosurgery in elderly patients with meningiomas.

## 2 Materials and methods

### 2.1 Study design and patient population

This is a retrospective analysis of a retrospectively and partly prospectively collected cohort (since 2020). The retrospective analysis and the registry for prospective patient data collection were approved by the local ethics committee as this cohort contained both datasets (ethical approval numbers: EA1/037/20, EA1/233/18). All patients in the prospective cohort signed a consent form. We identified and analyzed all patients over 60 years with suspected or histologically confirmed WHO grade I meningiomas who were treated with primary SRS at Charité University Hospital Berlin between January 2011 and December 2021. Patients who received re-irradiation for the same lesion were excluded from the study.

### 2.2 Data collection and analysis

Demographic, clinical, and radiographic data were retrospectively extracted and analyzed from clinical records and documentation. Tumor localizations were classified on preoperative MRI scans using “CLASS” algorithmic scale (26). Briefly, this classification is, in the context of meningiomas, devised for accurate classification and assessment of the spatial distribution of lesions based on their anatomical presence within the brain or surrounding structures. Tumor response was evaluated on the last available MRI scans of the patients using the Response Assessment in Neuro-Oncology (RANO) criteria for meningiomas (4). Therefore, the product of the transverse diameters of the lesion was calculated on the contrast enhanced T1 weighted MRI. To evaluate the safety of SRS, adverse effects were graded based on the Common Terminology Criteria for Adverse Events (CTCAE) version 5.0.

### 2.3 CyberKnife treatment

The indication for SRS treatment was made by an interdisciplinary team of neurosurgeons specialized in radiosurgery and radiation oncologists. The selected cases were discussed by an interdisciplinary neuro-oncological board. CyberKnife treatment was performed as previous described (14). Thermoplastic masks were individually produced and contrast-enhanced high-resolution thin-slice (0.75 mm) CT scans were performed. CT scans were co-registered with T1-weighted magnetic resonance scans (MRI: magnetization-prepared rapid acquisition with gradient echo using gadolinium-based contrast agents, 1.0 mm slice thickness). In selected cases with challenging anatomical locations, we performed Ga-68-DOTATOC PET-MRI or -CT to integrate this into planning, as described previously (14). The planning processes (dose prescription, fractionation scheme, target definition, and dose optimization) were performed by an experienced multidisciplinary team consisting of radiation oncologists, radiation physicists, and neurosurgeons. The gross tumor volume (GTV) was defined as the tumor volume based on contrast-enhanced CT and MRI. Planning target volume (PTV) margins of 0-1 mm were chosen by the treating physician in accordance with tumor morphology and location. Depending on the proximity to critical structures (organs at risk), such as optic nerves, the chiasm, the brainstem, and/or the size of the meningioma, various dose schedules were prescribed for a 70-85% isodose. In cases where the meningioma was situated close to an eloquent area (e.g., the brainstem or along the optic pathway), either a reduction in the dose per fraction or hypofractionation was implemented, depending on the fulfillment of dose constraints. The isodose volume of the normal brain tissue (NTB, excluding the PTV) for a single fraction (V10 Gy < 10cm^3^), three fractions (V18 < 10 cm^3^), and five fractions (V28.8 < 7 cm^3^) was calculated and noted in each patient as a standard clinical practice to ascertain the potential risk of adverse effects on the adjacent healthy brain tissue, as described previously (27). To protect other organs at risk, we applied the recommended threshold doses for SRS/HF-SRS published by Benedict et al. (28).

**Figure 1:**
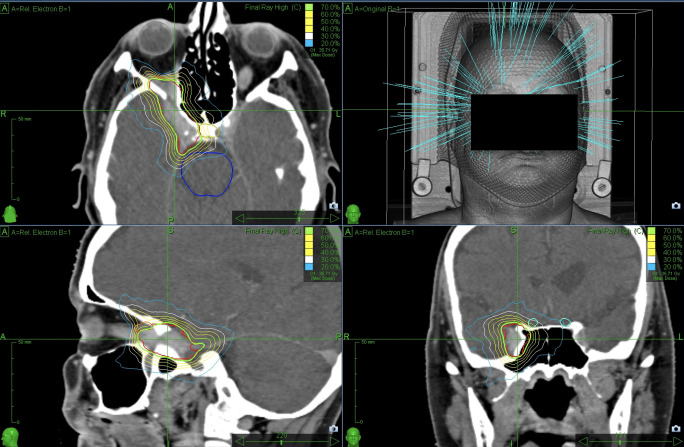
Exemplary CyberKnife planning representing a 61-year-old female patient suffering from a meningioma infiltrating the cavernous sinus. The green line marks the 70% isodose line surrounding the PTV (underlying red line which represent the contour by the physician). The lesion was irradiated in five fractions to a total dose of 25 Gray.

SRS was performed using a non-isocentric treatment approach that involved monitoring the patient’s skull approximately every 60 seconds using X-rays to ensure precise beam delivery. Each session was between 30 and 120 minutes long. An exemplary CyberKnife treatment plan for an included patient is shown in **Figure 1**.

Patients received dexamethasone 4 mg during or after treatment for 3–5 days, depending on the location and symptoms. In routine clinical practice, patients are invited for a follow-up examination 6 months after radiation therapy and thereafter at 6- or 12-month intervals for the first two years, and then annually.

### 2.4 Statistical analysis

Statistical analyses were performed using SPSS version 25 (IBM Corp.), RStudio Version 2023.06.1, Microsoft Excel 2021, and GraphPad Prism 8.4.2. Discrete data are presented as counts and percentages. Continuous data are presented as median and interquartile range [IQR]. The bidimensional product of diameters of the meningiomas prior to therapy and at follow-up were compared using the Wilcoxon matched pairs signed rank test; therefore, multiple meningiomas in the same patients were considered as independent lesions as the focus of this work relied not on confirming hypotheses but on exploring the data. Progression-free survival (PFS) was investigated using Kaplan–Meier analysis for local control lesions, also considering lesions independent. A progression was defined as progressive disease according to the RANO criteria. Patients were censored when follow-up was terminated prior to an event. Clinically relevant variables were selected and applied to a univariate Cox regression model to identify potential predictors. As none of the variables reached statistical significance, and because of the small absolute number of events, a multivariable analysis was not performed. In all analyses, two-sided p-values < 0.05 were taken to indicate statistical significance. However, owing to the exploratory nature of the study, no correction for multiple testing was applied, and p-values were given as an orientation and needed to be interpreted in a hypothesis-generating manner.

## 3 Results

### 3.1 Study population and treatment characteristics

A total of 82 patients with 102 lesions treated with SRS were enrolled in this study. The median patient age was 70 [IQR 64-75] years. Overall, 24.3% of the patients were aged > 75 years. A previous surgical procedure was performed in 57.3% of lesions with a median time interval of 41 [IQR 10-58] months between initial surgical resection and the SRS treatment. In these cases, a diagnosis of WHO grade I meningioma was confirmed histologically. Furthermore, 24.4% of the patients underwent a Ga-68-DOTATOC PET, indicating meningioma. The remaining patients were presumptively categorized as WHO grade I meningioma based on the course of the disease. Of the included patients, three (3.7%) had undergone previous cranial radiation therapy. None of the previous lesions (two meningiomas and one vestibular schwannoma) were at the same location as the current treatment site. The median bidimensional product prior to SRS-therapy was determined to 331 [IQR 163- 629] mm^2^. The median PTV of the lesions was observed to be 5.01 [IQR 2.26 – 9.93] cm^3^. Baseline characteristics of the study population are shown in **Table 1**.

**Table 1:** Baseline characteristics of the study population.

	Total study population (n= 82)
Age, yr, median [IQR]	70 [64 – 75]
Female sex, n (%)	55 (67.0)
KPS (%), median [IQR]	90 [60-100]
Surgery performed prior to CK-SRS, n (%)	34 (41.2)
CLASS classification	
CLASS 1, n (%)	22 (21.6)
CLASS 2, n (%)	30 (29.4)
CLASS 3, n (%)	50 (49.0)
Number of targeted lesions per patient per treatment	
1, n (%)	69 (84.1)
2, n (%)	9 (12.2)
3, n (%)	1 (1.2)
4, n (%)	1 (1.2)
6, n (%)	1 (1.2)
Tumor localization (n = 102)	
Skull base, n (%)	53 (52.0)
Convexity, n (%)	34 (33.3)
Optic sheath, n (%)	8 (7.8)
Falx or Tentorium, n (%)	7 (6.9)

CK-SRS, CyberKnife radiosurgery; IQR, Interquartile range; KPS, Karnofsky Performance Status; n, number; yr, years.

The majority of patients (84.1%) were presented with a single intracranial lesion. Concurrently, almost half (49.0%) of the study population showed a high-risk meningiomas according to the CLASS scale. The corresponding locations are detailed in **Supplementary Table 1**. In patients who had undergone previous surgery, the median duration between the last operation and the begin of SRS was 29 (IQR 9-75) months. Most patients included in this study exhibited favorable clinical conditions with minimal symptom-related impairments in daily activities prior to treatment. Karnofsky Performance Score (KPS) was 90-100 points in 61 (74.4%) patients, 70-80 points in 19 (23.2%) patients and < 70% in 2 (2.4%) patients. The corresponding treatment characteristics are provided in **Supplementary Table 1**. Briefly, most patients were treated with a single fraction (59.8%) of 14 or 15 Gy, whereas hypofractionation was performed in up to five fractions with a dose of 25 Gy or 27.5 Gy (see **Table 2**).

**Table 2:** Treatment characteristics.

	Lesions (n= 102)
**Single Session SRS, n (%)**	**61 (59.8)**
Prescription dose, Gy, median [IQR]	14 [14-15]
Isodose, %, median [IQR]	
Gross tumor volume, cm^3^, median [IQR]	3.0 [1.4-5.9]
Planning target volume, cm^3^, median [IQR]	3.0 [1.4-6.6]
**Hypofractionation, n (%)**	**41 (40.2)**
**3 Fractions, n (%)**	**10 (9.8)**
Dose, Gy, median [IQR]	21 [21-23]
Gross tumor volume, cm^3^, median [IQR]	11.3 [7.6-13.5]
Planning target volume, cm^3^, median [IQR]	12.6 [8.0-15.7]
**4 Fractions, n (%)**	**2 (2.0)**
Dose, Gy	20 and 22
Gross tumor volume, cm^3^	1.5 and 4.8
Planning target volume, cm^3^	1.5 and 4.8
**5 Fractions, n (%)**	**29 (28.4)**
Dose, Gy, median [IQR]	25 [25-25]
Gross tumor volume, cm^3^, median [IQR]	10.0 [3.8-14.4]
Planning target volume, cm^3^, median [IQR]	10.0 [3.8-16.3]

SRS, Stereotactic radiosurgery; IQR, Interquartile range; Gy, Gray; n, number.

### 3.2 Local control of lesions

Follow-up MRI scans were available for 69 (84.1%) patients for 87 (85.3%) lesions, with a median follow-up time of 15 [IQR 7 – 30] months after SRS. According to the RANO criteria for meningioma, most of these lesions (72.4%) were classified as stable disease in the latest follow-up scans, whereas 24.1% presented in a state of remission (minor to complete response). Response rates are summarized in **Table 3**.

**Table 3:** Tumor response based on the RANO criteria for meningiomas at the latest available follow-up (median follow-up time 15 [IQR 7 – 30] months).

	Lesions (n= 87)
**Complete response, n (%)**	**2 (2.3)**
Follow-up time, months	18 and 94
**Partial response, n (%)**	**4 (4.6)**
Follow-up time, months	8, 10, 15 and 20
**Minor response, n (%)**	**15 (17.2)**
Follow-up time, month, median [IQR]	30 [21-50]
**Stable disease, n (%)**	**63 (72.4)**
Follow-up time, month, median [IQR]	11 [6-28]
**Progressive disease, n (%)**	**3 (3.4)**
Follow-up time, months	5, 9 and 18

IQR, Interquartile range; n, number.

**Figure 2:**
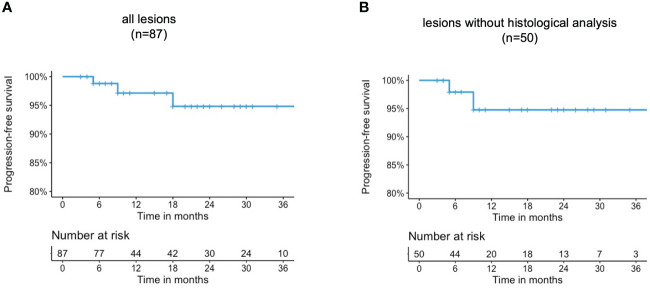
Progression-free survival rates of **(A)** all lesions **(B)** lesions without histological analysis.

**Figure 3:**
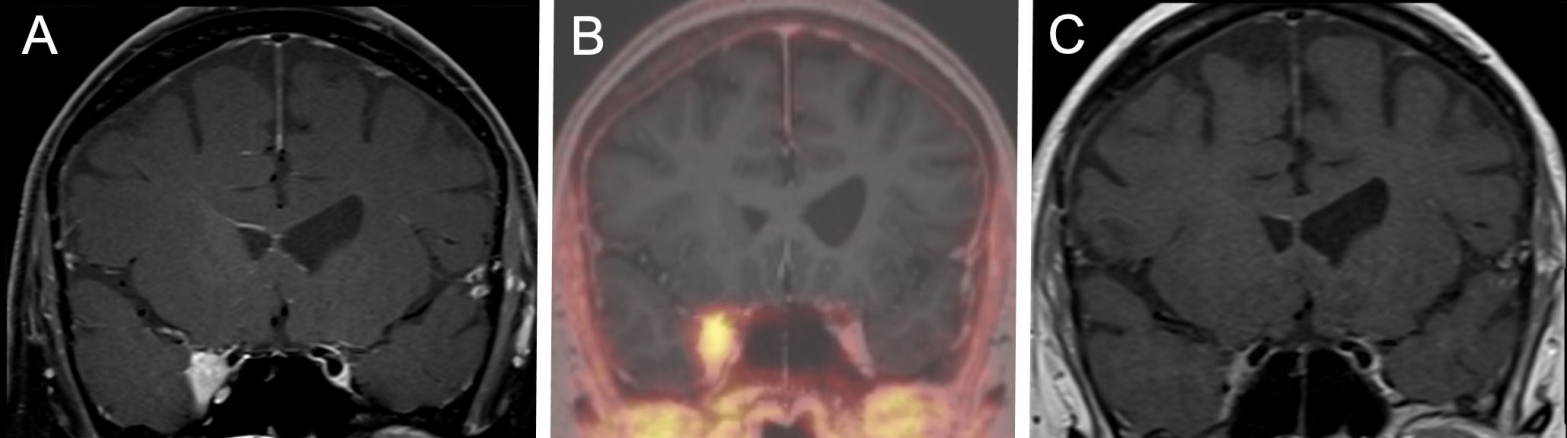
A representative case for the tumor response after hypofractionated SRS therapy. A 61-year-old female patient suffering from headaches and double visions. SRS was performed with a 5x5 Gray 70% isodose. **(A)** Coronal T1 weighted MRI scan after administration of Gadolinium prior to CyberKnife therapy showing a lesion infiltrating the cavernous sinus. **(B)** DOTATOC-PET/MRI Scan conforming a meningioma and enabling better target delineation. **(C)** Coronal T1 weighted MRI scan after administration of Gadolinium at the 18 months follow-up showing a complete remission of the lesion according to the RANO criteria. The patient reported a full remission of the headache and the double-visions at the follow-up.

The latest available follow-up examinations were assessed using Kaplan meier analysis. Accordingly, the estimated rates for local progression-free survival at 6, 12, and 24 months were 98.8% [95%CI 96.5-100], 97.1% [95%CI 93.2-100], and 94.8% [95%CI 89.1-100], respectively. The progression-free survival curves are shown in **Figure 2**. A complete response was observed in two patients after SRS therapy, each suffering from a single lesion. One of these patients was exemplary as presented in **Figure 3**. Three patients, each presenting with one intracranial lesion demonstrated notable increase in lesion size (159%, 170% and 144%) at the follow-up. Of these, two subjects had lesions located at the cerebral convexity, while the third patient’s lesion was situated at the optic sheath. Histological analysis was conducted in one of these lesions, confirming a WHO°1 meningioma. For the two other lesions no histological analysis was available.

**Figure 4:**
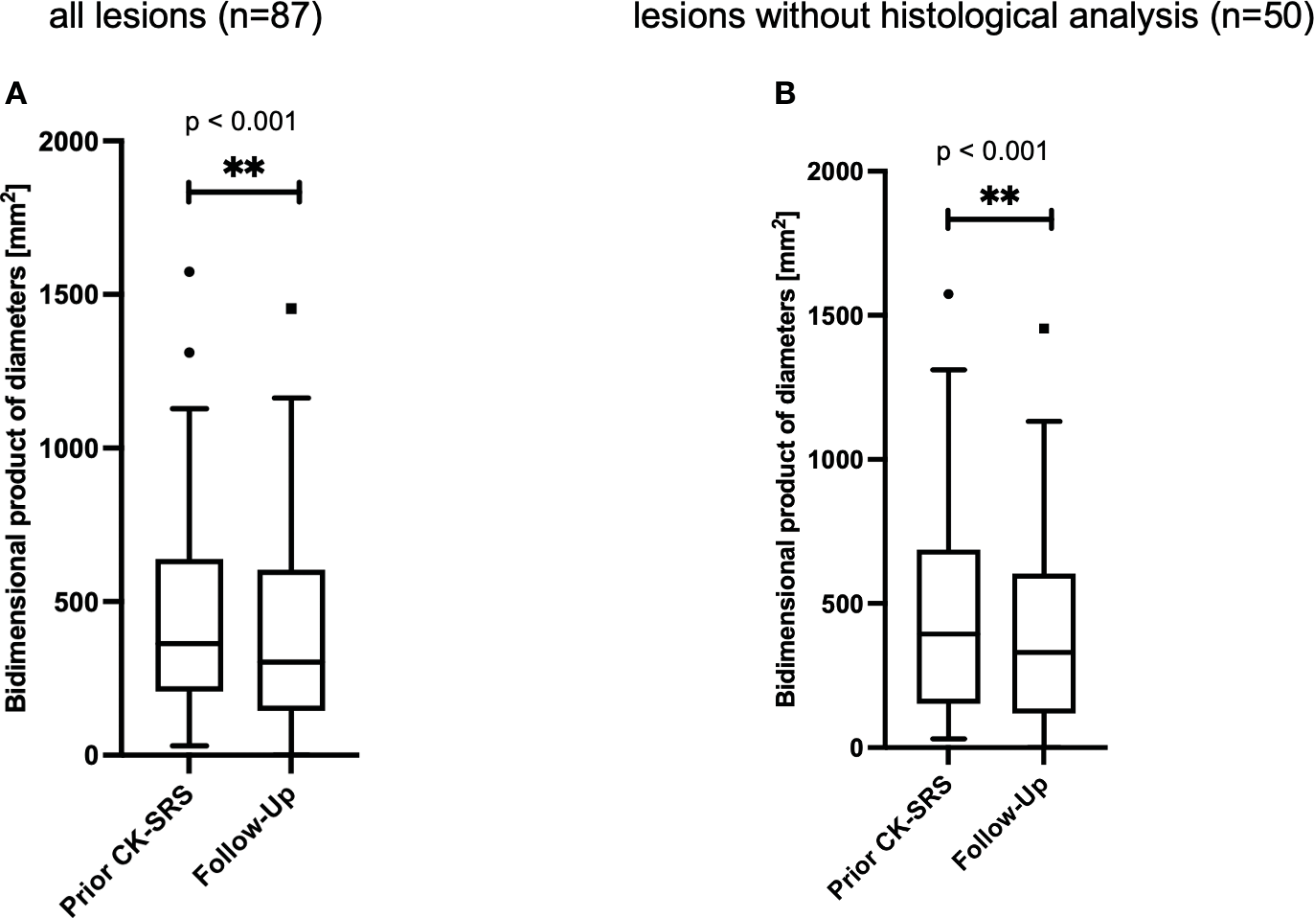
Bidimensional product of diameters of **(A)** all meningiomas (n=87 lesions in 69 patients). The median follow-up time was 15 [IQR 7 - 30] months. **(B)** all lesions without histological analysis (n=41 lesions in 35 patients). The median follow-up time was 11 [IQR 6- 23] months. Data is presented as Tukey-plot. **, significant (p<0.01).

To examine another aspect of lesion size changes, we additionally present the bi-dimensional product of diameter (BPD). Analysis of meningiomas demonstrated a reduction in lesion dimensions attributable to SRS therapy. The bidimensional product of diameters (BPD) for the 87 meningiomas under study decreased (p** < 0.001) from 364 mm^2^ [IQR 208–639] to 301 mm^2^ [IQR 144–604]. The median reduction in the BPD was observed to be 8.1% [IQR 2.7% – 23.6%]. Accordingly, a reduction of 0-10% was noted in 18 (20.7%) lesions, 10-20% in 11 (12.6%) lesions, 20-30% in 23 (14.9%) lesions, and more than 30% in 24 (16.1%) lesions (**Figure 4**).

### 3.3 Clinical outcome and complications

Clinical follow-up data were available for 71 patients, with a median duration to the most recent follow-up of 18 [IQR 8-35] months. Among these patients, the median KPS remained at 90 [IQR 80-90] points at the final clinical follow-up. Cumulatively, a maximum deterioration in KPS of 20 points was noted in four patients, representing 5.6% of this cohort. The latest follow-up periods for these four patients were 4 months (lesion in the cavernous sinus), 11 months (petroclival lesion), 41 months (lesion at the convexity) and 94 months (parasagittal lesion). An improvement in the KPS score (10 points each) was observed in five patients (follow-up times: 12, 14, 37, 40, and 78 months; locations: clival, clinoidal, tuberculum sellae, petroclival, and petrous parts of the temporal bone).

Complications were reported in 13 patients (18.3%) with available clinical follow-up data. Among these patients, 9 (69.2%) experienced a mild reversible complication of CTCAE grade 1, 3 (23.1%) experienced a CTCAE grade 2 complication (vestibular disorder, seizure, dysarthria due to edema; all not persistent), and one patient was affected by a grade 3 complication (trigeminal neuralgia; persistent). Importantly, this complication was the only persistent morbidity in this cohort.

To assess whether there were predictors of complications, we performed univariate Cox proportional hazards regression analyses for age, planning target volume, surgery before CK-SRS, and lesion location. Regression analysis revealed no predictors of complications. The results are presented in **Table 4**.

**Table 4:** Potential predictors of complications (univariate cox regression)

	HR (95% CI)	p-value
Age	1.007 (0.919-1.03)	0.883
Planning target volume	1.035 (0.961-1.15)	0.355
Surgery prior to CK-SRS	0.788 (0.216 – 2.874)	0.718
CLASS 3	0.846 (0.334 – 3.808)	0.846

CI, Confidence interval; CK-SRS, CyberKnife radiosurgery; HR, Hazard Ratio.

## 4 Discussion

This study investigated the safety and local control rates of SRS in elderly patients with meningioma. This pooled retrospective/prospective analysis of 82 patients with 102 SRS-treated lesions revealed a low incidence of high-grade adverse events (four patients with CTCAE ≥2) and excellent local control during the median follow-up period of 15.9 months. These findings suggest that SRS is a safe and effective treatment option for elderly patients with meningiomas, demonstrating favorable outcomes in terms of both safety and local control, even in high-risk locations. SRS may therefore prevent potential neurological deficits due to tumor growth.

An increase in the incidence of meningiomas in the elderly population can be anticipated owing to ongoing demographic transition towards an older society (2, 29). Highly specialized treatment plans are sometimes limited to younger patients in routine clinical practice, while older patients are more likely to receive a watch-and-wait treatment. However, because meningiomas can become symptomatic and may cause mass effects, it is important to offer appropriate treatment to older patients. Our data reveals sufficient tumor control through non-invasive SRS therapy in this patient cohort. In only three (3.4%) of the meningiomas in this study a progression at the follow-up could be detected. We formally recorded these as progressive diseases, resulting in an estimated local control rate of 97.1% after 12 months. However, all these lesions had short follow-up periods within the first year, implying that the observed progression could be a case of pseudoprogression, and volume reduction could still be seen in future radiological assessments. The occurrence of transient enlargement in meningiomas and acoustic neurinomas mainly in the first year after SRS has recently been reported, highlighting the critical interpretation of early progression in benign intracranial diseases (30–33). Accordingly, transient enlargement of the tumor may be attributed to pseudoprogression in response to SRS and should be initially observed if asymptomatic. A further analysis of the course of the disease beyond the cutoff date of this study was performed. Two of the patients also showed a further progress (follow-up time: 13 months and 14 months), resulting in one of these patients undergoing surgical treatment 14 months after SRS. Importantly, this must also be interpreted with caution, as it also does not ensure whether it was a true progression since it was still in the early period after SRS; however, it is important to note that a possible pseudoprogression can also cause neurological symptoms and require surgery. In routine clinical practice, it is of great importance that no further treatment is indicated in asymptomatic patients in the early phase, as the transient enlargement of benign tumors after SRS is now well known. No additional follow-up data were available for the third patient. Moreover, the histological grade of these lesions may not be I, an aspect that cannot be investigated in non-operated cases.

The vast majority of the meningiomas (72.4%) showed a stable behavior according to the RANO criteria without significant volume changes, whereas 24.1% were classified as mild to complete response (remission). This finding is consistent with the results of a comparable study that investigated the incidence of pseudoprogression in meningiomas after stereotactic radiosurgery (30). An analysis of the bidimensional products of meningioma diameters revealed a significant reduction in the lesion mass after SRS therapy. We explain the difference between significant mass reduction in the analysis of the diameters and mainly stable disease according to the RANO criteria by the fact that a reduction of up to 25% is still considered as stable disease. Within the cohort of lesions examined, 43.6% showed a reduction of at least 10% at the follow-up, with a median of 15 months after SRS. Particularly in critical locations, this decrease could have clinical relevance and bring about improvements in the patient. Although long-term data are fundamentally important for meningiomas, given the advanced age of the patients included in this study, the follow-up horizon of the present study represents a clinically significant time span. The tumor control reported in our study is consistent with comparable studies (2, 34, 35). Kaul et al. (2) retrospectively investigated tumor control after image-guided stereotactic radiotherapy for meningiomas in 98 elderly patients. In this series, 93.7% of the patients with a median age of 71 years showed a stable disease at the 36 months follow-up. Fokas et al. (35) analyzed 121 patients with a median age of 73 years who were treated with fractionated stereotactic radiotherapy and showed local tumor progression only in 4.9% lesions, at a median follow-up time of 40 months, similar to our data.

Our data suggest an overall safe treatment option for elderly patients. In this study, we reported no mortality and a morbidity rate of 18.3%. Thereby, only 5.6% patients showed severe complications (CTCAE ≥ 2) and only one of these complications (1.4%) was persistent. This is particularly compelling, as 49.0% of the included patients were treated for lesions at high-risk locations (CLASS 3). Comparable studies of elderly patients suffering from intracranial meningiomas in diverse locations who were treated with surgery showed higher morbidity rates from 21 to 30% (15, 29, 36, 37). Limited data are available on the complication rates after radiotherapy. Kaul et al. (2) reported rates of 54.1% with acute toxicity and 16.3% with chronic toxicity after image-guided stereotactic radiotherapy in elderly patients suffering from meningiomas (2). Our Cox regression analyses revealed no potential risk factors for complications in our patients. To the best of our knowledge, there are no current risk factors for complications in elderly patients treated with SRS. Particularly, we examined the role of high-risk localization, which has not yet been confirmed as a risk factor. However, owing to the overall low number of complications in this study, further subgroup analysis was not feasible. Thus, further studies on the risk factors are warranted.

This study has several limitations. First of all, the present study was conducted in a single tertiary center providing advanced specialty care to a population of approximately 4 million inhabitants. Therefore, bias due to the single-center design could not be ruled out. Furthermore, only patients exhibiting favorable clinical conditions were selected for treatment with SRS due to the outpatient setup of our center. This is reflected by the initial high median KPS of 90 in the cohort. This presents a potential bias. The implications of this therapeutic approach on individuals with compromised functionality remain undetermined and were not addressed within the scope of this study. Additionally, by design, this study is intrinsically constrained by its pooled retrospective analysis. Moreover, the study is inherent limited due to the lack of a comparison group. Therefore, comparative statements regarding groups of younger patients, of patient groups untreated with SRS or treated with surgery cannot be made. The fact that not all patients had histological results available is a limitation of the study. These patients were categorized as WHO grade I meningioma inferred from the disease progression observation. Importantly, the course of the lesions after SRS confirms the benign pathology as grade I tumors. Due to the lack of a sufficient number of preoperative MRI scans, the study cannot make assertions regarding the volume reduction of previous surgery. Moreover, with a median follow-up duration of 15 months, extended monitoring results remain forthcoming for the evaluation of potential late recurrence of meningiomas. However, with regard to the age of the patients, the follow-up period was reasonable. Owing to the small number of lesions and events, we confined the variables for the Cox regression analysis to four, aiming to identify potential predictors of complications. Consequently, the scope of the study was constrained, precluding the examination of additional potentially interesting variables.

## 5 Conclusion

In conclusion, our data indicate that, in short-to mid-term, SRS is a safe and effective treatment option for elderly patients with meningiomas. The study findings demonstrated favorable outcomes in terms of safety, with a low incidence of adverse events and excellent control rates. These results support SRS as a promising therapeutic approach for managing meningiomas in the elderly population, even in high-risk locations to avoid clinical deterioration as a result of tumor progression. We propose that SRS does not substitute surgical intervention but presents as an alternative for smaller or anatomically challenging lesions especially for potentially multimorbid elderly patients in whom surgery is not a viable option or can be performed as part of a hybrid treatment. Future prospective studies with larger cohorts and longer follow-up times are warranted to confirm our findings.

## Data availability statement

The raw data supporting the conclusions of this article will be made available by the authors, without undue reservation.

## Ethics statement

The studies involving humans were approved by Ethikkommission der Charité – Universitätsmedizin Berlin Campus Charité Mitte Charitéplatz 1 (Virchowweg 10) 10117 Berlin (ethical approval numbers: EA1/037/20 and EA1/233/18). The studies were conducted in accordance with the local legislation and institutional requirements. The participants provided their written informed consent to participate in this study.

## Author contributions

AF: Conceptualization, Data curation, Formal analysis, Investigation, Methodology, Visualization, Writing – original draft, Writing – review & editing. BB: Data curation, Formal analysis, Validation, Writing – review & editing. MN: Formal analysis, Writing – review & editing. JG: Data curation, Writing – review & editing. GK: Data curation, Writing – review & editing. KR: Formal analysis, Methodology, Validation, Writing – review & editing. PT: Formal analysis, Writing – review & editing. DK: Formal analysis, Methodology, Supervision, Validation, Writing – review & editing. DZ: Resources, Supervision, Validation, Writing – review & editing. PV: Formal analysis, Project administration, Resources, Supervision, Validation, Writing – review & editing. CS: Conceptualization, Formal analysis, Funding acquisition, Investigation, Methodology, Project administration, Resources, Software, Supervision, Validation, Visualization, Writing – review & editing. GA: Conceptualization, Formal analysis, Funding acquisition, Investigation, Methodology, Project administration, Resources, Software, Validation, Visualization, Writing – review & editing.
